# Safely catching aerial micro-robots in mid-air using an open-source aerial robot with soft gripper

**DOI:** 10.3389/frobt.2022.1030515

**Published:** 2022-11-02

**Authors:** Zhichao Liu, Caio Mucchiani, Keran Ye, Konstantinos Karydis

**Affiliations:** Department of Electrical and Computer Engineering, University of California, Riverside, Riverside, CA, United States

**Keywords:** aerial systems, biologically-inspired robots, soft robots, grasping, manipulation planning, field robots

## Abstract

This work focuses on catching safely an aerial micro-robot in mid-air using another aerial robot that is equipped with a universal soft gripper. To avoid aerodynamic disturbances such as downwash, that would push the target robot away, we follow a horizontal grasping approach. To this end, the article introduces a gripper design based on soft actuators that can stay horizontally straight with a single fixture and maintain sufficiently compliance in order to bend when air pressure is applied. Further, we develop the Soft Aerial Gripper (SoAG), an open-source aerial robot equipped with the developed soft end-effector and that features an onboard pneumatic regulation system. Experimental results show that the developed low-cost soft gripper has fast opening and closing responses despite being powered by lightweight air pumps, responses that are comparable to those of a commercially available end-effector tested we test against. Static grasping tests study the soft gripper’s robustness in capturing aerial micro-robots under aerodynamic disturbances. We experimentally demonstrated the feasibility of using the SoAG robot to catch a hovering micro-robot with or without propeller guards. The feasibility of dynamic catching is also shown by capturing a moving aerial micro-robot with a velocity of 0.2 m/s. The free flight performance of the SoAG robot is studied against a conventional quadrotor and in different gripper and payload status.

## 1 Introduction

Grasping with aerial robots attracts increasing interest from both research institutes and companies across industry sectors, owing to these robots’ unique capability to operate in 3-dimensional (3D) space while avoiding terrain constraints that often limit access to ground robots (and humans) Ruggiero et al. (2018). Grasping can be defined as a sequence of three key consecutive steps: 1) approaching a target, 2) establishing contact with the object, and 3) securing and holding the object firmly Meng et al. (2021). Grasping is also a crucial ability for aerial robots to interact with the environment and facilitate several key applications such as inspection Yüksel et al. (2015), search and rescue Gómez-de Gabriel et al. (2018), transportation Fiaz et al. (2018), and construction Augugliaro et al. (2014).

The most common way to achieve aerial grasping is to directly mount robotic manipulators onto appropriate aerial robots. Notable examples include multirotor aerial vehicles with mostly servo-driven robotic arms Korpela et al. (2013); Jimenez-Cano et al. (2013); Ruggiero et al. (2015); Garimella and Kobilarov (2015); Baizid et al. (2015); Kim et al. (2016); Zhang et al. (2018); Staub et al. (2018). To overcome payload limitations, unmanned helicopters have been utilized to carry industry manipulators Pounds et al. (2011a); Kondak et al. (2013); Bejar et al. (2019). Aerial robots have also been equipped with dual robotic arms for precise manipulation Korpela et al. (2014); Suarez et al. (2017) as well as parallel manipulators Danko and Oh (2013); Fumagalli et al. (2014); Danko et al. (2015). Multi-link robotic arms can provide precise position control of the end-effector with improved reachability. However, mounting robotic arms on aerial robots requires larger scales thereby leading increasing costs and compromising mobility in confined environments. Such aerial manipulators often employ ordinary multi-finger end-effectors; studying the ability to grasp irregularly-shaped micro-objects has received less attention. A fixed-wing aerial vehicle is equipped with a passive claw for high-speed grasping Stewart et al. (2022). However, the robot is still constrained to grasping regular objects such as poles. In a different line of work, multi-robot systems can be leveraged to grasp and move objects Ritz et al. (2012); Zhao et al. (2017); Gabrich et al. (2018); Shi et al. (2020). However, cooperative grasping increases the computational effort on control and planning, and requires significant system integration efforts to be practical. The aerial grippers are often constrained to vertical grasping, which limits potential applications.

Distinctly from rigid aerial robot grasping, soft (compliant) grasping has been receiving increased attention due to its advantages of being robust and safe to irregularly-shaped objects Shintake et al. (2018). Several micro aerial vehicles (MAVs) have been equipped with soft (compliant) end-effectors, including but not limited to impactive and ingressive Mellinger et al. (2011), compliant multi-fingered Ghadiok et al. (2011); Pounds et al. (2011b); Kruse and Bradley (2018); McLaren et al. (2019); Zhang et al. (2019); Appius et al. (2022); Chen G. et al. (2022), closed-structure compliant Lee et al. (2021), origami-inspired Kim et al. (2018), wasp-pedal-carrying Zhao et al. (2018), soft cable-driven Ramon-Soria et al. (2019); Fishman et al. (2021), and soft pneumatic Mishra et al. (2018) grippers. However, these aerial grippers have been limited to vertical grasps directly underneath the robot, which, besides limiting applicability, is also impacted by aerodynamic disturbances.

In recent years, there is a growing interest in developing non-military tools to capture aerial robots in mid-air with applications to recover malfunctioning aerial robots and intercept and contain unidentified flying targets Park et al. (2021). Physically catching flying robots in mid-air is challenging due to their irregular shapes and self-propulsion. Notable attempts include a soft gripper fixed on a ground manipulator to catch flying micro-robots Fedoseev et al. (2021). However, the solution is limited by vertical grasping, as well as the workspace of the ground manipulator. The most common way to catch flying robots in mid-air is using nets, such as net bullets Meng et al. (2018); DroneCatcher (2022), top nets Rodriguez-Ramos et al. (2021); DroGone (2022), side nets Vidyadhara et al. (2022), and nets carried by cooperative vehicles Klausen et al. (2018); Rothe et al. (2019). Despite the proved effectiveness, these solutions primarily focus on catching aerial robots with diagonal sizes (including propellers) over 500 mm (e.g., DJI1 Mavic Pro and Phantom 4). However, little attention is paid to capturing flying micro-robots such as Crazyflie2 2.1 with diagonal sizes around 100 mm, which are relatively more challenging to detect and intercept Park et al. (2021). In addition, capturing by nets involve relative motion to targets that will inevitably create impact and possibly damage target robots. Flying robots with nets are usually unable to grasp, move and release objects compared to ones with conventional grippers. A recent work studies catching aerial micro-robots with a passive gripper Chen T. G. et al. (2022). However, the capture relies on contact with the propeller guard of the target, which is usually missing with commercially available aerial vehicles. After capturing, the suspending target will compromise the free fly performance of the catcher Kim et al. (2016). Similarly, the method requires relative velocities to the target, and creates impact.

**FIGURE 1:**
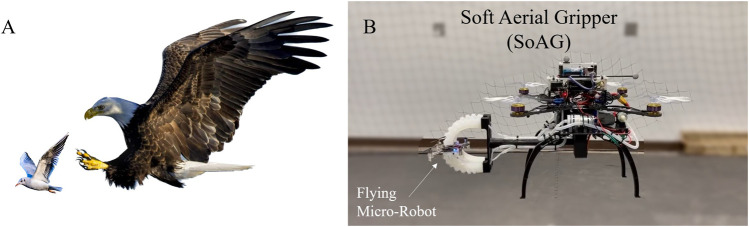
Bio-inspiration and prototype of the project. **(A)** An eagle is horizontally catching a small bird in mid-air. **(B)** To enable horizontal grasping, we develop an open-source micro-aerial vehicle (MAV) equipped with a soft end-effector and onboard pneumatic regulation named Soft Aerial Gripper (SoAG).

In this work, we aim to address the challenge of catching aerial micro-robots safely in mid-air using flying robots with a universal soft gripper. To this end, we introduce a soft actuator and pneumatic four-fingered end-effector designs to enable horizontal grasps. As shown in Figure 1, we develop a quadrotor MAV equipped with a soft end-effector named Soft Aerial Gripper (SoAG). The hardware design of SoAG is introduced, as well as the dynamic modeling and control. Piecewise-polynomial-based optimal planning is studied to facilitate catching of flying targets. Static grasping results are compared to a commercially-available gripper to validate the efficacy of grasping irregular objects. Experimental trials also demonstrate the feasibility of using the SoAG robot to catch a target aerial micro-robot while both agents are flying without relative velocities to mimimize impact. We study the robustness of the soft gripper with catching tests of flying targets with or without propeller guards. Furthermore, we study the feasibility of dynamic catching by capturing a moving aerial micro-robot in the mid-air. Lastly, free flight performance of the SoAG robot is studied and compared to a conventional quadrotor to validate the design and evaluate the effect of the gripper on flight mechanics, control, and energetics. The project is open-sourced to facilitate rapid replication of the developed robot.

## 2 Materials and methods

### 2.1 Design

This project exploits soft robotic grippers to catch flying micro-robots safely. However, horizontal grasps are challenging for most soft grippers because they cannot stay horizontally straight with a single fixture. As mentioned earlier, horizontal captures are critical to avoid aerodynamic disturbance (downwash), which can make targets crash. Thus, this paper revises the Pneumatic Network (PneuNet) design Polygerinos et al. (2013); Mosadegh et al. (2014) to enable horizontal grasps.

#### 2.1.1 Actuator design and fabrication

**FIGURE 2:**
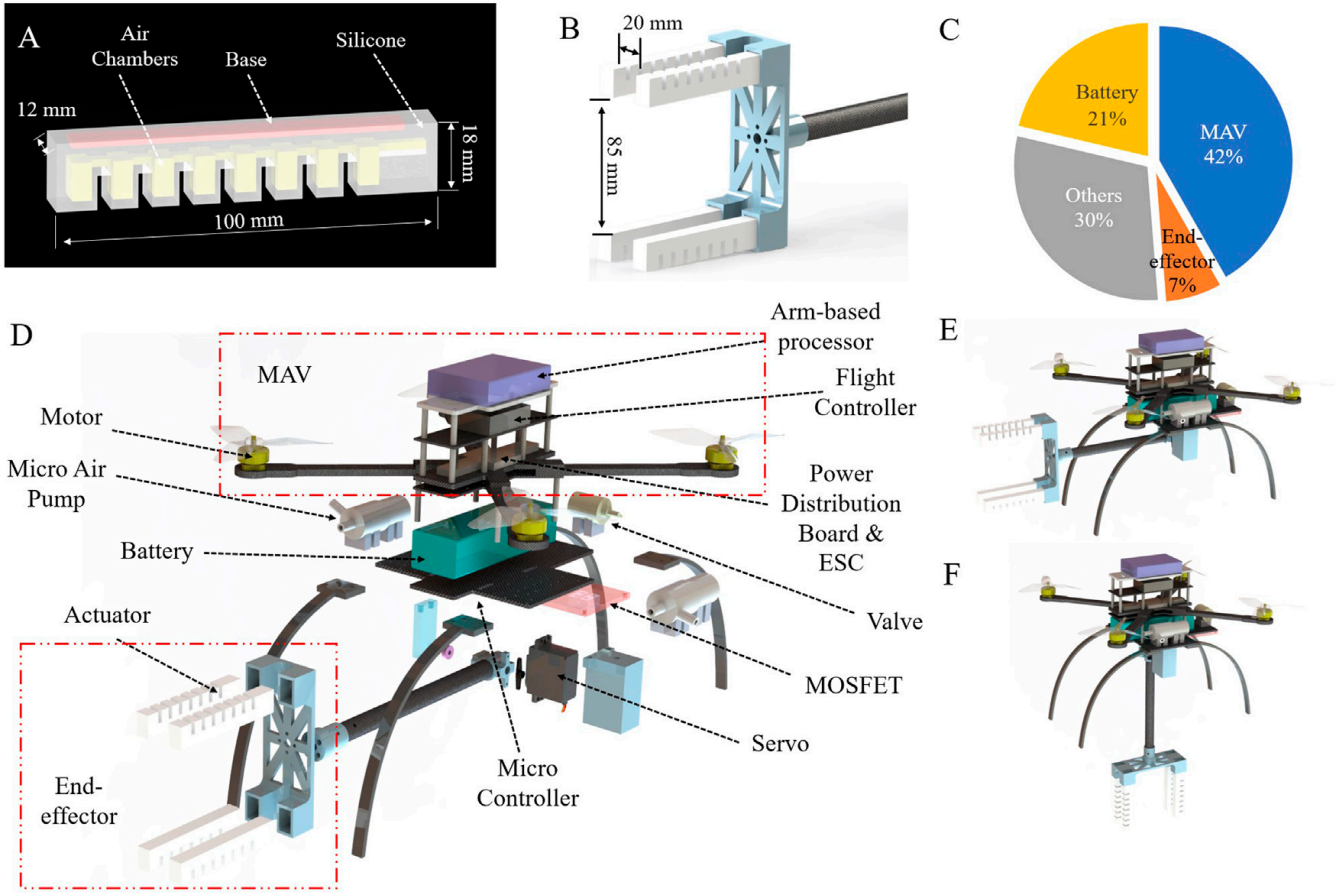
Details of the system design. **(A)** Actuator design. **(B)** End-effector design. **(C)** Weight distribution of the SoAG robot. **(D)** An exploded view of the SoAG robot. **(E,F)** The robot with the arm up and down.

Two improvements to the PneuNet design are made to achieve horizontal grasps. To minimize deformation by gravity and keep the softness for safe interactions, appropriate stiffness of the actuators is achieved by combining two materials with different shore hardness. As shown in Figure 2A, the main body (white) is made of Smooth-On Dragon Skin 20 silicone with shore hardness of 20A (A here meaning the type A indenter and scale) to maintain the softness for adaptive grasps. In the PneuNet design, there is an inextensible layer to assist bending, which is made of thin fabric or paper Polygerinos et al. (2013). However, the thin inextensible layer fails to increase the stiffness sufficiently for horizontal grasps. In this work, we replace the thin inextensible layer with a solid flexible cuboid base (part shown in red in Figure 2A), which is 3D-printed using the Formlabs flexible 80A resin. The base has a shore hardness of 80A. Despite increased hardness, the actuators still maintain enough compliance to bend relatively fast under pneumatic inflation. Moreover, the actuator’s width is reduced and base thickness is increased to accelerate actuation response and support the horizontal-grasping potential. Details of the revised dimensions can be found in Figure 2A, where parts in yellow denote the implanted air chambers.

The fabrication of actuators follows the conventional method of casting with molds. The custom molds are 3D-printed in polylactic acid (PLA) while bases are directly 3D-printed with flexible 80A resin as mentioned earlier. With molds and bases ready, we mix the elastomer and process it using a degassing chamber. After cured, the two casted parts of the actuator (chamber and base layers) are bonded with an adhesive (Sil-Poxy). Note that the flexible 80A base should be surrounded by silicone in the manufacturing of the base layer. We open-source all mold design files to enable rapid replication of the actuator.

#### 2.1.2 End-effector design

Similar to conventional grippers, our four-fingered soft end-effector consists of two opposing claws. When inflated, the tips come together on opposite sides (top and bottom) of flying micro-robots to grasp them. Each claw of the gripper has two actuators in parallel with a gap of 20 mm while the two opposite sides have a distance of 85 mm as shown in Figure 2B. All actuators are fixed by a 3D-printed adapter connecting to the aerial platform using a carbon fiber rod. The end-effector weighs 0.115 kg, accounting for only 7% of the total weight (see Figure 2C). Similarly, we open-source all files to fabricate the end-effector. The total cost of fabrication is about *$*40. We evaluated the end-effector performance by comparing it to a commercially available gripper, as detailed in Section 3.

#### 2.1.3 Soft aerial gripper robot design

We mount the end-effector on a custom quadcopter MAV to develop the SoAG robot. The robot has a total weight of 1.64 kg, which consists of four types of components: MAV, battery, end-effector, and other parts (see Figure 2C). A hardware overview of SoAG is shown in Figure 2D. The custom-made MAV features frames that are fabricated with lightweight carbon fiber sheets (tensile strength 120, 000–175, 000 psi) using a Stepcraft D.600 CNC router with enclosure and milling bath. The MAV measures 380 mm from the motor tip to tip. It integrates a flight controller (Pixhawk 4 Mini) running the corresponding open-source autopilot system. The vehicle also includes an ARM-based multi-core processor (Odroid XU4) for high-level computing tasks.

In addition to the MAV and end-effector (highlighted in red boxes in Figure 2D), the robot also includes pneumatic regulating components necessary to power the soft gripper. The onboard pneumatic regulation consists of two micro air pumps, one solenoid valve, and one MOSFET module. The air pumps have a flow rate of 2.0 L/min with a low weight of about 0.07 kg. When the robot tries to catch a target, the two pumps will inflate four actuators to bend (one pump per two actuators) and close the gripper. All actuators are also connected to the normally-closed solenoid valve, of which the other side is directly open to the atmosphere air. When pumps are off and the valve is on, the pressure values inside the actuators will decrease to the atmospheric one so that the gripper will open. The MOSFET module reads PWM signals and regulates the DC voltages of the pumps and valves. SoAG has one revolute joint to move the position of the end-effector to the main robot (see Figures 2E,F). A MG 996R servo motor controls the angle of the revolute joint between the vehicle and the arm. We use a micro-controller (Arduino Nano) to control both the pneumatic actuation and the arm angle.

### 2.2 Modeling

**FIGURE 3:**
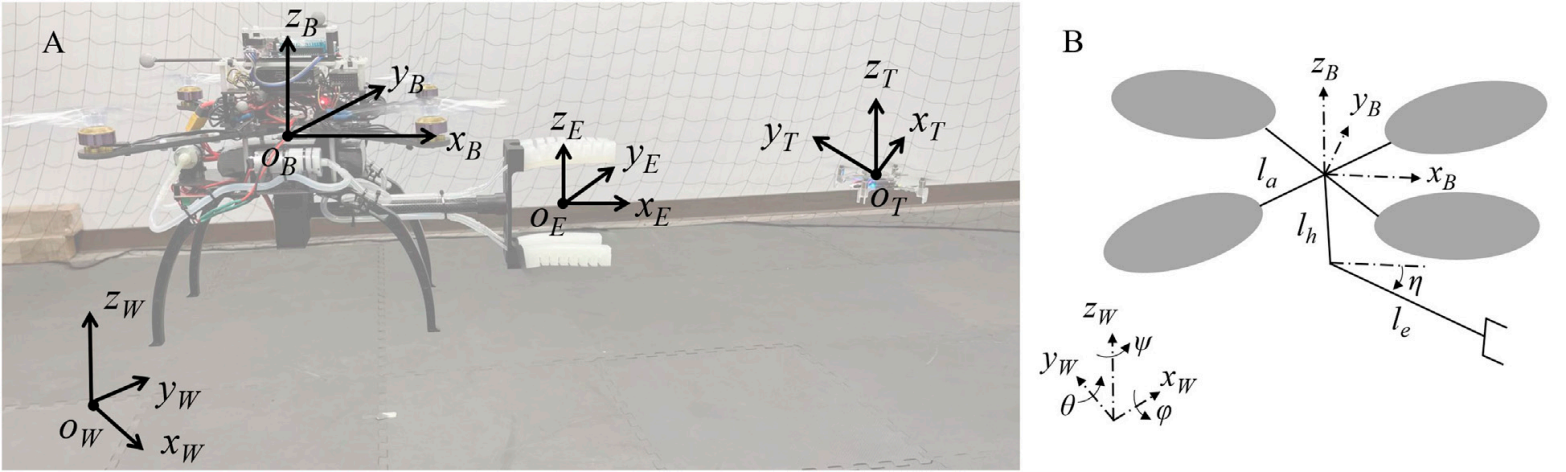
Frames and modeling description. **(A)** Four frames are defined in this work: world, robot, end-effector and target frames. **(B)** Dynamic model of the system.

We considered NWU (X North, Y West, Z Up) as the world frame, denoted with $W:\left\{O_{W};x_{W},y_{W},z_{W}\right\}$ (see Figure 3A). The body frame of the robot is denoted with $B:\left\{O_{B};x_{B},y_{B},z_{B}\right\}$ ; its origin coincides with the robot’s center of mass. We also define the end-effector frame E with its origin at the center of the gripper. Also let $T:\left\{O_{T};x_{T},y_{T},z_{T}\right\}$ be the frame attached to the target. We use $R_{WB}\in \text{SO}\left(\text{3}\right)$ to denote the orientation of the body frame B in the world frame W . $R_{WB}$ can be written following the Z-X-Y sequence as

$$R_{WB}=\left[\begin{array}{ccc} c_{\phi }c_{\psi }-s_{\phi }s_{\theta }s_{\psi } & -c_{\theta }s_{\phi } & c_{\phi }s_{\psi }+c_{\psi }s_{\phi }s_{\theta }\\ c_{\psi }s_{\phi }+c_{\phi }s_{\theta }s_{\psi } & c_{\phi }c_{\theta } & s_{\phi }s_{\psi }-c_{\phi }c_{\psi }s_{\theta }\\ -c_{\theta }s_{\psi } & s_{\theta } & c_{\theta }c_{\psi } \end{array}\right]\;,$$ (1)

where *c* denotes the cosine, *s* stands for sine, and Euler angles *ϕ*, *θ*, and *ψ* denote rotating angles along the axis *x*, *y* and *z*, respectively.

The generalized coordinate variables comprise the position of $O_{B}$ $\left(p={\left[x,\;y,\;z\right]}^{T}\in R^{3}\right)$ , the Euler angles $\left(\Phi ={\left[\phi ,\;\theta ,\;\psi \right]}^{T}\in R^{3}\right)$ of the aerial robot in the world frame, as well as the joint angle η∈R with respect to the zero position as in Figure 3B. For simplicity, we drop the superscript W for the world frame. The vector that contains all the generalized coordinate variables can be written as $\xi ={\left[\;p^{T},\;\Phi^{T},\;\eta \;\right]}^{T}\in R^{7}$ .

As shown in Figure 3B, the revolute joint lies along the axis $z_{B}$ and its distance from the robot’s center of mass $O_{B}$ is *l*_*h*_. Let *l*_*e*_ be the length between the joint and the center of the end-effector $O_{E}$ . The arm of the end-effector is constrained within the $x_{B}-z_{B}$ plane of the body frame. Thus, we can find the position of $O_{E}$ ( $p_{E}\in R^{3}$ ) in the world frame as.

$$R_{BE}=\text{Rot}\left(y_{B},\eta \right)=\left[\begin{array}{ccc} c_{\eta } & 0 & s_{\eta }\\ 0 & 1 & 0\\ -s_{\eta } & 0 & c_{\eta } \end{array}\right]$$ (2a)

$${}^{B}pE={\left[\;0,\;0,\;-l_{h}\;\right]}^{T}+R_{BE}{\left[\;l_{e},\;0,\;0\;\right]}^{T}$$ (2b)

$$={\left[\;l_{e}c_{\eta },\;0,\;-l_{h}-l_{e}s_{\eta }\;\right]}^{T}$$ (2c)

$$p_{E}=p+R_{WB}\;{}^{B}pE.$$ (2d)

Using the Euler-Lagrange formulation, we can derive the equations of motion as

$$\begin{array}{cc} \frac{d}{dt}\frac{\partial L}{\partial \dot{\xi }}-\frac{\partial L}{\partial \xi } & =F=\left[\begin{array}{c} 0\\ 0\\ f\\ \tau \\ \tau_{\eta } \end{array}\right]\in R^{7}\\ L & =K-U \end{array}$$ (3)

where f∈R is the total thrust along $z_{B}$ axis, $\tau ={\left[\tau_{x},\;\tau_{y},\;\tau_{z}\right]}^{T}\in R^{3}$ includes the torque vector generated by the four motors, and $\tau_{\eta }\in R$ is the torque of the revolute joint. The kinetic K and potential U energy of the system are functions of the generalized coordinate variables. For the kinetic energy we have

K=KB+KE,KB=12mBp˙Tp˙+12ωBTIBωB,KE=12mEp˙ETp˙E+12ωEBTRBEIERBETBωE, (4)

where $m_{B}$ and $m_{E}$ stand for the mass of the main body and end-effector, respectively. The arm that connects to the end-effector has very small mass (less than 10 g) and it is hence excluded from the overall dynamics calculations. The velocity of the end-effector ${\dot{p}}_{E}$ can be found by taking the derivative of Eq. 2d. Here, ${}^{B}\omega \in R^{3}$ denotes the angular velocity of the main robot in the body frame while ${}^{B}\omega \in R^{3}$ stands for the end-effector angular velocity in the body frame. Both angular velocities can be related to the generalized coordinate variables as

$$\begin{array}{cc} {}^{B}\omega & ={\left(R_{WB}\right)}^{T}T\dot{\Phi },\\ {}^{B}\omega E & =J_{E}\dot{\eta }, \end{array}$$ (5)

with $T\in R^{3\times 3}$ being the transformation matrix such that $\omega =T\dot{\Phi }$ , and $J_{E}\in R^{3\times 1}$ relating the angular velocity of the end-effector in the body frame to the manipulator’s joint angle. The potential energy U can be calculated as

$$U=m_{B}g\;z_{W}^{T}\;p+m_{E}g\;z_{W}^{T}\;p_{E},$$ (6)

where $z_{W}={\left[0\;0\;1\right]}^{T}$ denotes the unit vector along *z* axis in the world frame and *g* is the gravity constant.

By combining the equations above, we can rewrite the dynamic modeling of the entire system as

$$M\left(\xi \right)\ddot{\xi }+C\left(\xi ,\dot{\xi }\right)\dot{\xi }+G\left(\xi \right)=F$$ (7)

where $M\left(\xi \right)\in R^{7\times 7}$ is the inertia matrix, $C\left(\xi ,\dot{\xi }\right)$ is the Coriolis matrix and *G*(*ξ*) includes gravitational forces. Readers are referred to Kim et al. (2016) for details about calculating these matrices.

### 2.3 Control

**FIGURE 4:**
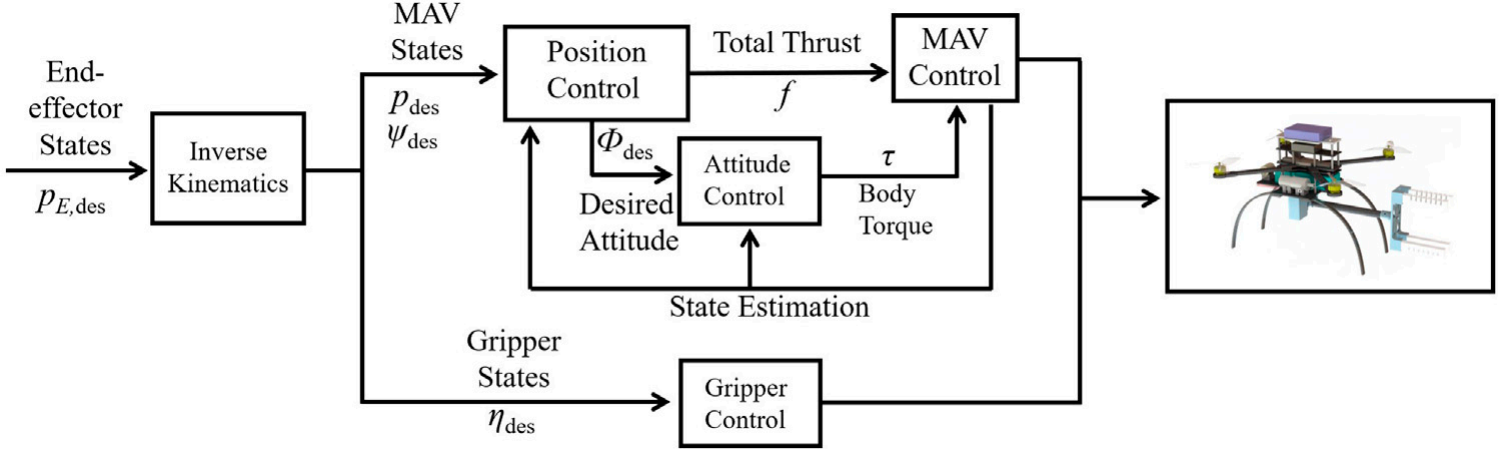
A cascaded tracking controller is used in this work to regulate both the vehicle and gripper.

As detailed in Figure 4, the controller reads desired states of the end-effector $p_{E,\text{des}}$ from the planner, which will be elaborated in Section 2.4. From Eqs 2a, 2b, 2c, 2d, $p_{E,\text{des}}$ is decided by the desired states of MAV *p*_des_ and gripper *η*_des_. Note that both *p*_des_ and *η*_des_ are free variables that can change the end-effector position. We opt to fix the desired angle of the gripper $\left(\eta_{\text{des}}=0\;\text{or}\;\frac{\pi }{2}\right)$ . The orientation of the MAV $R_{WB}$ also affects Eq. 2d. Considering the differential flatness of the MAV system Sreenath et al. (2013), the controller uses a constant desired yaw angle *ψ*_des_ = 0 while the desired roll *ϕ*_des_ and pitch *θ*_des_ angles will be calculated based on the desired and actual states of the robot.

To eliminate the assumption of small angles near hovering states, we adopt a nonlinear cascaded tracking control method based on geometric constraints as in Lee et al. (2010); Mellinger and Kumar (2011); Thomas et al. (2016). As shown in Figure 4, the cascaded control method includes position and attitude controllers. The position controller reads the desired position *p*_des_, velocity ${\dot{p}}_{\text{des}}$ , acceleration ${\ddot{p}}_{\text{des}}$ and yaw angle *ψ*_des_, and outputs total thrust f∈R in body frame and desired attitude $\Phi_{\text{des}}\in R^{3}$ . The attitude control reads the desired and actual attitude and outputs body torque *τ* as in Eq. 3. Given the desired and actual states of the MAV, we can find the desired force vector $F_{\text{T,des}}\in R^{3}$ in the world frame

$$F_{\text{T,des}}=-K_{d}\left(\dot{p}-{\dot{p}}_{\text{des}}\right)-K_{p}\left(p-p_{\text{des}}\right)+m{\ddot{p}}_{\text{des}}+mgz_{W},$$ (8)

where $K_{d},K_{p}\in R^{3\times 3}$ are diagonal, positive definite tuning matrices. We can find the desired total thrust *f* in body frame

$$f=F_{\text{T,des}}^{T}\cdot z_{B}=F_{\text{T,des}}^{T}\cdot \left(R_{WB}^{T}z_{W}\right).$$ (9)

Since the aerial vehicle can only generate thrust along the $z_{B}$ axis, the desired $z_{B,\text{des}}$ direction is aligned with *F*_*T*,des_; the $y_{B,\text{des}}$ direction is chosen to match the desired yaw *ψ*_des_. Thus, the desired attitude $R_{\text{des}}\in SO\left(3\right)$ is calculated as

$$\begin{array}{cc} z_{B,\text{des}}= & \frac{F_{T,\text{des}}}{\left\|F_{T,\text{des}}\right\|}\\ a_{\psi }= & {\left[\cos\psi_{\text{des}},\;\sin\psi_{\text{des}},\;0\right]}^{T}\\ y_{B,\text{des}}= & \frac{z_{B,\text{des}}\times a_{\psi }}{\left\|z_{B,\text{des}}\times a_{\psi }\right\|}\\ R_{\text{des}}= & \left[y_{B,\text{des}}\times z_{B,\text{des}},\;y_{B,\text{des}},\;z_{B,\text{des}}\right] \end{array}$$ (10)

where the operator × denotes the cross product. Note that a singularity exists when calculating $y_{B,\text{des}}$ . Readers are referred to Thomas et al. (2016) to address the singularity problem. With the desired yaw *ψ*_des_ directly from the planner, the desired attitude in Euler angles $\Phi_{\text{des}}={\left[\phi_{\text{des}},\;\theta_{\text{des}},\;\psi_{\text{des}}\right]}^{T}$ can be calculated based on the Z-X-Y sequence as

$$\begin{array}{cc} R_{\text{des}}= & \left[\begin{array}{ccc} R_{11} & R_{12} & R_{13}\\ R_{21} & R_{22} & R_{23}\\ R_{31} & R_{32} & R_{33} \end{array}\right]\\ \phi_{\text{des}}= & \text{arctan}\left(-\frac{R_{12}}{R_{22}}\right)\\ \theta_{\text{des}}= & \text{arctan}\left(\frac{R_{32}}{\sqrt{1-R_{32}^{2}}}\right) \end{array}$$ (11)

We adopt a nonlinear attitude control method as in Brescianini et al. (2013). The attitude controller reads the estimated actual and desired attitude, and outputs the desired angular velocity to the low-level PID bodyrate controller. The nonlinear controller is asymptotically stable, and readers are referred to the report Brescianini et al. (2013) for a thorough analysis. The low-level bodyrate controller is implemented in the open-source PX4 firmware Meier et al. (2015).

### 2.4 Planning

Aerial micro-robots are vulnerable to aerodynamic disturbances (e.g., downwash and ground effect) generated by other aerial vehicles Preiss et al. (2017) or rigid surfaces Karydis et al. (2015); Karydis and Hsieh (2016). Thus, planning for the catching task seeks to generate smooth trajectories that satisfy catching constraints, without producing downwash effect that may destabilize the target aerial robot and while remaining out of ground effect regions that depend on the robot size, propeller length and forward velocity Kan et al. (2019). Trajectory generation for aerial robots has been extensively studied (e.g., Hehn and D’Andrea, (2011); Kreciglowa et al. (2017); Mohta et al. (2018)). The planner generates smooth desired trajectories for the end-effector based on piecewise polynomials as in Mellinger and Kumar (2011); Richter et al. (2016). Assuming the path has *m* segments (and therefore we have *m* + 1 key frames to apply constraints *t* ∈ {*t*_0_, *t*_1_, … , *t*_*m*_}), we use *n*th order polynomial functions to describe the segment *i* on axis *μ* ∈ {*x*, *y*, *z*}, considering the desired yaw always set to zero (*ψ*_des_ = 0). That is,

$$\sigma_{\mu ,i}\left(t\right)=c_{\mu ,i}^{T}\left[\begin{array}{c} 1\\ t\\ \vdots \\ t^{n} \end{array}\right],\;t\in \left[t_{i-1},t_{i}\right],$$ (12)

where $c_{\pi ,i}\in R^{n+1}$ contains coefficients of the polynomial segment. Thus, the desired trajectories can be found by optimizing the objective function

$$J=\underset{\mu \in \left\{x,y,z\right\}}{\sum }\int_{t_{0}}^{t_{m}}\|\frac{d^{k}\sigma_{\mu ,i}\left(t\right)}{dt^{k}}{\|}^{2}dt.$$ (13)

Following the minimum-snap formulation Mellinger and Kumar (2011), we minimize the snap along the trajectory, so *k* = 4 and *n* = 8. Then, the trajectory generation can be reformulated as a quadratic program

$$\begin{array}{cc} \text{min} & \;c^{T}Hc\\ \text{s.t.} & \;Ac\leq b, \end{array}$$ (14)

where $c\in R^{3m\left(n+1\right)\times 1}$ that contains all polynomial constants can be found as

$$c_{i}=\left[\begin{array}{c} c_{x,i}\\ c_{y,i}\\ c_{z,i} \end{array}\right]\;c=\left[\begin{array}{c} c_{1}\\ c_{2}\\ \vdots \\ c_{m} \end{array}\right].$$ (15)

**FIGURE 5:**
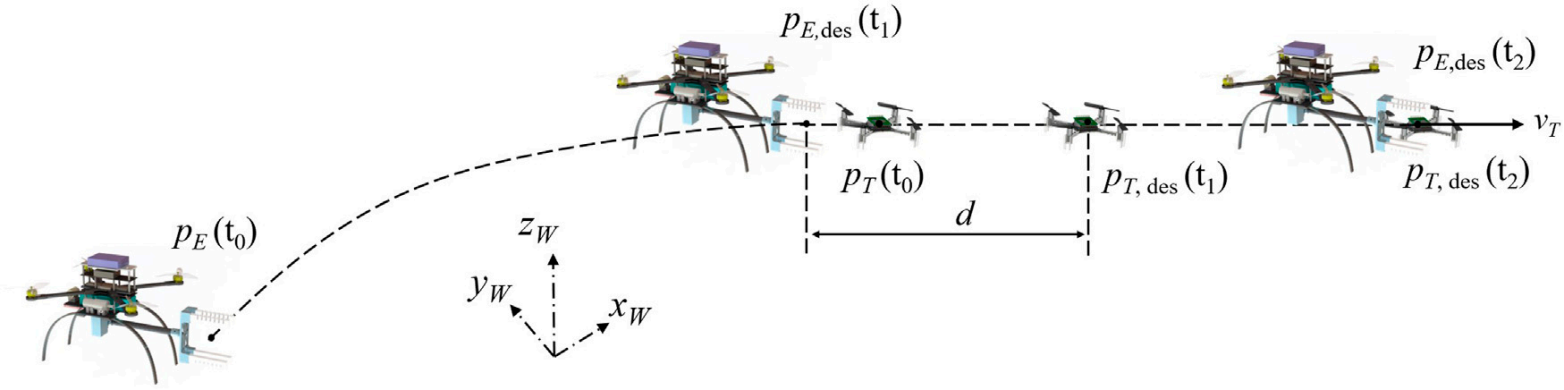
Piecewise-polynomial-based planning for a sample catching scenario.

The constraint *Ac* ≤ *b* in Eq. 14 is described next. As shown in Figure 5, the target aerial robot is assumed to follow a constant velocity $v_{T}={\dot{p}}_{T}$ in the world frame. The position of the target is measured as $p_{T}\left(t_{0}\right)$ in the world frame at time *t*_0_. Projected positions of the flying target are denoted as $p_{T,\text{des}}\left(t\right)$ at time *t* based the constant velocity assumption. We use $P_{E}\left(t_{0}\right)$ to denote the initial gripper position where *t*_0_ is the starting time. Similar to related work Rodriguez-Ramos et al. (2021), we separate a catching task into three segments (chase, close and grasp), with three key frames to apply optimization constraints (*t*_1_ and *t*_2_). In the chase part (*t* ∈ [*t*_0_, *t*_1_]), the end-effector of the robot tracks a trajectory from $P_{E}\left(t_{0}\right)$ to the position $P_{E,\text{des}}\left(t_{1}\right)$ , which lies along the direction of $v_{T}$ with a distance of d∈R .

$$\begin{array}{cc} P_{E,\text{des}}\left(t_{1}\right)= & P_{T,\text{des}}\left(t_{1}\right)-R_{WT}\left(d\frac{v_{T}}{\left\|v_{T}\right\|}\right),\\ P_{T,\text{des}}\left(t_{1}\right)= & P_{T}\left(t_{0}\right)+\left(t_{1}-t_{0}\right)v_{T},\\ {\dot{P}}_{E,\text{des}}\left(t_{1}\right)= & v_{T}. \end{array}$$ (16)

In the close segment (*t* ∈ [*t*_1_, *t*_2_]), the end-effector moves from $P_{E,\text{des}}\left(t_{1}\right)$ to the projected position of the flying target $P_{E,\text{des}}\left(t_{2}\right)=p_{T,\text{des}}\left(t_{2}\right)$ with the constant velocity ${\dot{P}}_{E,\text{des}}\left(t_{2}\right)=v_{T}$ . At time *t*_2_, the end-effector is automatically triggered to start inflating to grasp the target. The key frame *t*_1_ is calculated as

$$t_{1}=t_{0}+\alpha \|P_{T}-P_{E}\left(t_{0}\right){\|}_{2},$$ (17)

and *t*_2_ = *t*_1_ + *τ*_1_, *t*_3_ = *t*_2_ + *τ*_2_, where *α*, $\tau_{1},\tau_{2}\in R$ are constants.

## 3 Results

Results are categorized based on three types of tests: grasping, catching, and flyability. In the grasping test, the soft gripper is studied against a commercially available gripper, mounted on a Kinova Gen3-lite robot for response and static catching tests. In the catching experiment, the target aerial robots hovers at a fixed position with tracking errors. After taking off manually, the developed SoAG robot generates and tracks a trajectory automatically to catch the flying target as described in Section 2.4. Finally, we study the free flight tracking performance of the robot with different arm and gripper states compared to a conventional quadrotor.

All experiments rely on motion capture camera systems (VICON and OptiTrack) for odometry feedback. The feedback is only used to estimate the states of the robots, which can also be achieved by cameras or laser sensors in outdoor environments. The Crazyflie 2.1 with MoCap deck is used as the target aerial robot, with a total weight of 0.035 kg. The developed SoAG robot measures 0.38 m from the motor tip to tip, with a total weight of 1.64 kg. A 3-cell 5200 mAh LiPo battery is used to power the entire system. Key parameters for different tasks can be found in Table 1.

**TABLE 1:** Key parameters of the developed SoAG robot.

$m_{B}$	$m_{E}$	*l*_*e*_ (m)	*l*_*h*_ (m)	*d* (m)	*α*	*τ*_1_ (s)	*τ*_2_ (s)
1.526 kg	0.115 kg	0.32	0.14	0.5	2	1.5	2

### 3.1 Grasping

**FIGURE 6:**
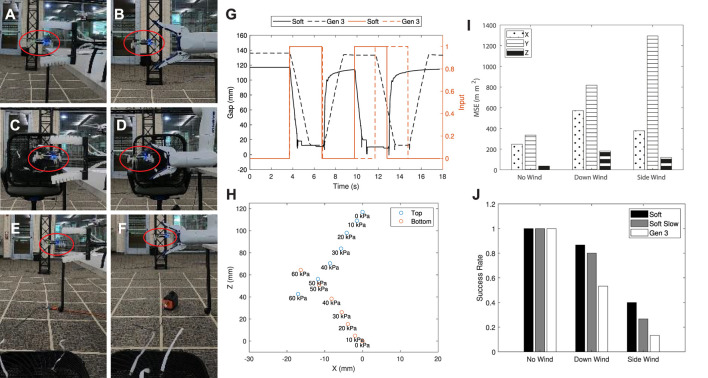
Grasping test for the soft and Gen3-lite grippers. **(A–F)** Both grippers are placed vertically at the same 3D position and studied in three cases: no-wind, down-wind, and side-wind. **(G)** Step response for both grippers. **(H)** Relative positions of the soft gripper’s two claws with respect to different pressure values. **(I)** Mean squared error for a hovering micro-robot under disturbances. **(J)** Success rates for horizontal grasping of a flying micro-robot using both grippers.

In this experiment, both grippers are placed vertically at the same 3D position, as shown in Figures 6A,B. The position of both claw tips is measured using the OptiTrack motion capture feedback in 100 Hz. We use gaps in millimeter to denote the position difference along the $z_{W}$ axis. Figure 6G presents the result of the response test, where black solid and dashed curves denote the gaps of the soft and Gen3-lite grippers, respectively. Similarly, orange solid and dashed curves stand for the input signals for the soft and Gen3-lite grippers. The input value of 1 means both grippers close (inflate) at the fastest speed. For the input value of 0, the valve opens to enable ventilation of the actuators for the soft gripper, and for the gripper mounted on the Gen3-lite, it opens at the fastest possible speed.

As shown in Figure 6G, we command the soft gripper to close and open for 3 s, while the Gen3-lite gripper is controlled to close for 3 s and open for 5 s. The Gen3-lite gripper has an initial opening of 136 mm, larger than the one of the soft gripper (117 mm). However, the soft gripper completely closes after 0.72 s, while it takes 1.90 s for Gen3-lite gripper to do so, with closing velocities 162.50 and 71.58 mm/s for the soft and Gen3-lite grippers, respectively. Large amounts of noise are observed when the soft gripper completely closes. The noise comes from the rigid-body-based motion capture system model, and the fact that inflated soft actuators have shape changes which introduce measuring errors. On the other hand, unlike the same speeds of the Gen3-lite gripper, the soft gripper has a much faster response for opening, with only 0.24 s to reach 85% of the initial gap. The normally-straight actuators with the flexible 80A support recover very fast with basic ventilation. Admittedly, the response of both grippers can be improved by having more powerful motors or inflators. However, the low-cost soft gripper introduced in this work has good performance powered by lightweight air pumps, compared to commercially available end-effectors like the Gen3-lite gripper.

Second, positions of the soft gripper’s two claws (top and bottom) are studied with respect to different pressure values 3. The end-effector is placed vertically along $z_{W}$ with the gap facing $-x_{W}$ , while the origin is located at the bottom claw in absence of pressurization. Figure 6H shows relative positions of top (blue) and bottom (red) claws when pressurized separately. The results indicate that the top claw has larger vertical deformation than the bottom one with the same pressure value due to gravity. However, both claws have much smaller horizontal deformation than the vertical one. The results also indicate the gripper completely closes when the pressure value reaches 50 kPa.

In the catching test, both grippers aim to grasp an aerial micro-robot that is hovering. In addition to irregular shapes, micro aerial robots can have comparably larger tracking errors in the hover state, thus making it a challenge to grasp by grippers. To further investigate the robustness of catches, three cases are studied in this test: no-wind, down-wind, and side-wind. As shown in Figures 6C–F, the target aerial micro-robot hovers under disturbance in down-wind and side-wind modes. Specifically, we use a fan to create aerodynamic disturbances at a distance of 1 m along $z_{T}$ and $y_{T}$ axis in down-wind and side-wind cases, respectively.

Figure 6I presents the mean squared error (MSE) between actual and desired hovering positions in three cases. Results show that the hovering micro-robot has larger tracking errors under these disturbances, especially along $y_{T}$ axis. Fifteen grasping trials are conducted for each gripper in each case, and all success rates are visualized in Figure 6J. Both grippers have good performance in the no-wind case. However, the soft gripper shows advantages in both down- and side-wind cases, owing to its ability to adapt to different shapes. Both grippers struggle in grasping in the side-wind case, due to the large tracking errors and grippers’ limited reachability along $y_{T}$ axis. To study the individual contributions of both the softness and closing velocity, one additional case (Soft Slow) is studied when we slow the closing speed of the soft gripper to 71.58 mm/s by outputting only 0.88% of the maximum voltage (10.56/12 V) to air inflators during pressurization. Results show that the soft gripper has similar grasping performance in the down-wind case with a reduced closing speed, however, the success rate drops when the disturbance rises in the side-wind case, supporting the significance of a fast closing speed.

Lastly, we study the maximum force applied by both grippers to validate the catching safety. A digital force gauge is used to measure vertical force along $z_{W}$ axis applied by the top claws of both grippers. Results show that the Gen 3-lite gripper has a maximum grasping force of 23.7 N while the soft gripper can only produce forces up to 0.63 N, which result in safe interactions with aerial targets. In the meantime, the developed soft gripper is experimentally proven able to grasp and hold (both horizontally and vertically) irregular objects such as multi-meters, pressure gauges and game controllers, with masses up to 280 g. The test validates the developed soft gripper can grasp most aerial micro-robots, as well as other irregular objects to function as a universal gripper.

### 3.2 Catching

#### 3.2.1 Static

In this test, we study the feasibility of using the developed SoAG robot to catch an aerial micro-robot that is hovering. The target $p_{T}$ hovers at the position [1.0, 1.0, 1.0]^*T*^ with tracking errors as in Figure 6G. The target’s position and velocity information are available to the catcher *via* motion capture feedback at all times. The end-effector of the catcher $p_{E}$ starts with an initial position [−0.68, 0, 1.0]^*T*^. The catcher robot generates and follows trajectories as in Section 2.4 with parameters listed in Table 1.

**FIGURE 7:**
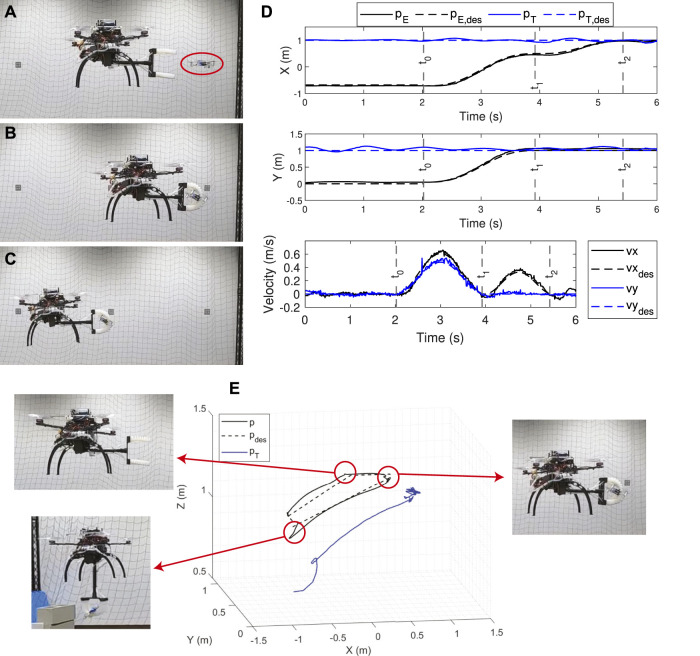
A sample trial of the static catching test. **(A–C)** The SoAG robot approaches the target, grasps the target, and returns with the target. **(D)** State tracking for the end-effector and target. **(E)** 3D positions of the SoAG robot and the target aerial robot.

A sample trial is presented in Figure 7, where images depict events when the SoAG robot approaches the target (A), grasps the target (B), and returns (C) with the target. Figure 7D shows the state tracking for the end-effector and target, where black solid and dashed curves denote the actual and desired positions of the end-effector in $x_{W}$ and $y_{W}$ directions. Similarly, blue solid and dashed curves represent the actual and desired positions of the target. The bottom figure shows the actual and desired velocities of the end-effector along $x_{W}$ and $y_{W}$ axis. The time gap *τ*_1_ is calculated per Eq. 17. Since *τ*_2_ is a constant, key frames to apply constraints are found as *t*_1_ = 3.92 s, *t*_2_ = 5.42 s and *t*_3_ = 7.42 s with a starting time *t*_0_ = 2.02 s. The figure demonstrates the good tracking performance of the SoAG robot with the planned smooth trajectory. The results also show the target has very small tracking errors before and during the grasping, which supports the claim that horizontal grasps do not produce aerodynamic disturbances detrimental to the target’s stability.

The actual and desired 3D positions of the SoAG robot are plotted in Figure 6E, as well as the target’s 3D position. The robot has larger tracking errors during the chase segment, and smaller errors for the close part. The results back up the planning method in piecewise polynomials to achieve small tracking errors before the grasping. Note that random noise is still present in the target’s hovering position, which makes aerial catching more challenging. Owing to the robustness of the soft gripper, the catcher robot manages to grasp the target and return with it. At the end of the trajectory, SoAG rotates the arm to place the target at a lower position and gets ready to drop the target safely.

**FIGURE 8:**
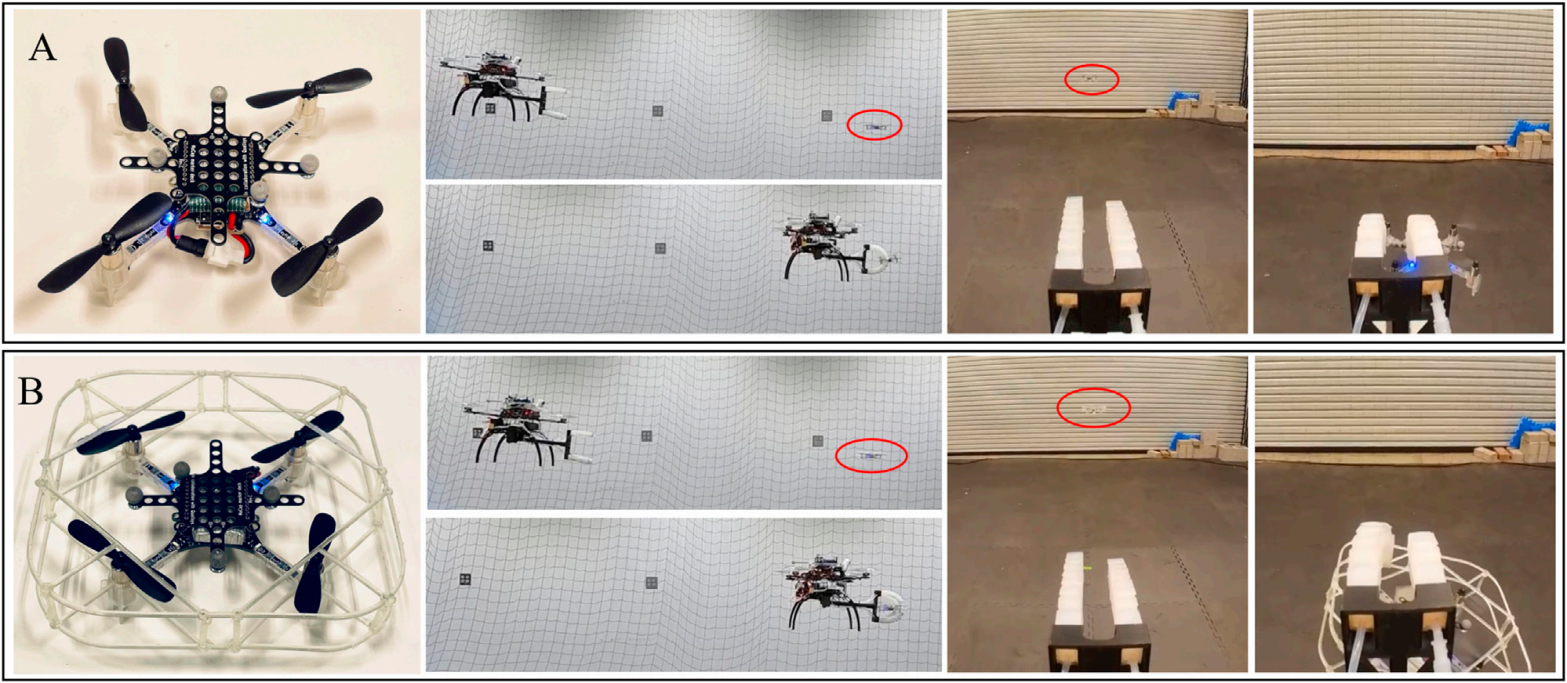
Close-up images of the flying targets without **(A)** or with **(B)** propeller guards, as well as side views and first-person views of the aerial captures.

While the related work Chen T. G. et al. (2022) relies on the contact with the propeller guards, our method can catch flying micro-robots with or without protective frames. In this part, we study the robustness of the soft gripper by introducing the catching test of flying targets with propeller guards. Figure 8 shows close-up images of the flying targets used in the tests with (B) or without (A) propeller guards. The flying micro-robot with the protective frame has a dimension of 130 × 130 × 40 mm, and a total mass of 40 g. The custom propeller guard is 3D-printed using the Form 3 in clear resin, which has the post-cured ultimate tensile strength 65 MPa. We repeat the static catching tests on flying targets, and the robot can successfully capture hovering micro-robots regardless of the propeller guards. Figure 8 presents side and first-person views of the aerial catching in both cases, where red ellipses mark hovering micro-robots. As shown in first-person views, the robot can catch flying targets even though the gripper is not in align with the center of the target, thanks to the robustness of the soft grasping. In the meantime, the observation that aerial catching does not damage the fragile propeller guards further support the safety of our method.

#### 3.2.2 Dynamic

In this test, we study the feasibility of dynamically catching an aerial micro-robot that is following a path. The flying target takes off at the position [−1.0,0,1.0]^*T*^ and moves along $x_{W}$ with a constant velocity of 0.2 m/s. The SoAG robot hovers at the position [−2.0,0,1.0]^*T*^ before the dynamic catching is manually triggered. The robot reads the actual position and velocity of the target *via* motion capture feedback at time *t*_0_ and plans the trajectory as described in Section 2.4.

**FIGURE 9:**
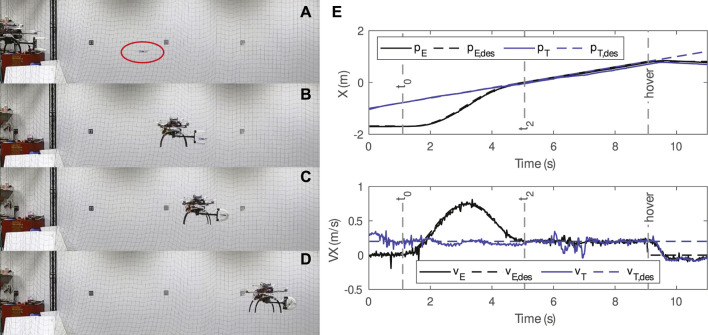
A sample trial of the dynamic catching test. **(A–D)** The SoAG robot chases, reaches and grasps the moving aerial target. **(E)** State tracking for the end-effector and moving target. Note that the planning skips the chase segment due relatively close starting positions.

A sample dynamic catching trial is shown in Figure 9, where the SoAG robot triggers the catching (A), starts inflating (B), finishes grasping (C), and enters hovering state (D). Figure 9E visualizes the actual and desired states tracking of both the catcher and target robots. Due to the limited space of the experimental area, the catcher robot hovers at a position relatively close to the flying target, thus the planning skips the chase segment. The results show that the planner generates a smooth trajectory for *t* ∈ [*t*_0_, *t*_2_] = [1.1, 5.2] to reach the same position and velocity of the target. At time *t*_2_, the SoAG robot starts inflating the end-effector to grasp the target, and keeps the constant velocity for another 4 s before the hovering state.

The position profile in Figure 9E shows that the robot is tracking the desired trajectory well. After the grasping, the target’s position has a small deviation from the desired one. Both the catcher and target robots remain the same position during the hovering. On the other hand, velocities tracking shows that the target has relatively larger tracking errors compared to the catcher, especially after the grasping. The catcher robot follows the smooth desired velocities well to reach and maintain the target velocity $v_{T}$ before the hovering state. The dynamic catching test supports the robot’s potential applications to rescue or intercept moving aerial targets. Compared to the related work Chen T. G. et al. (2022), our method can capture moving aerial targets while staying relatively static to minimize impact.

### 3.3 Flyability

**FIGURE 10:**
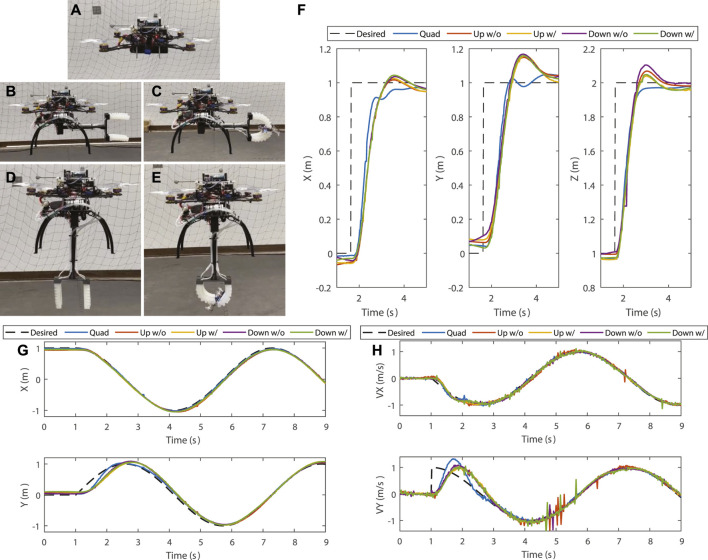
Free flight tracking test. **(A–E)** Five cases are considered in the test: conventional quadrotor (Quad), SoAG robot with arm up without target (Up w/o), SoAG robot with arm up with target (Up w/), and SoAG robot with arm down without target (Down w/o). **(F)** States for all robots in the step response test. **(G)** Positions and **(H)** Velocities on $x_{W}$ and $y_{W}$ axis for the planar circle tracking test.

In this experiment, we study the effect of the arm and target on the free flight tracking performance of the catcher. As shown in Figures 10A–E, five cases are considered in the test: conventional quadrotor (Quad), SoAG with arm up and without target (Up w/o), SoAG with arm up and seized target (Up w/o), SoAG with arm down and without target (Down w/o), and SoAG with arm down and seized target (Down w/). The angle *η* = 0 when the arm is up, and *η* is $\frac{\pi }{2}$ for the arm-down case. Note that Quad (conventional quadrotor built in-house without any gripper) has a total weight of 1.035 kg including the battery while the target’s weight is 35 g. Due to a smaller weight, tuning parameters are different for Quad, while other four cases share all variables. The experiment comprises two parts: step response and planar circle tracking. In the step response test, all robots hover at the point [0,0,1]^*T*^ before the planner sends discrete setpoints [0,0,2]^*T*^, [1,0,2]^*T*^ and [1,1,2]^*T*^ at 5 s intervals. For the second test, all robots track a planar circle trajectory centered at the point [0,0,1]^*T*^ with a radius of 1 m. The circle starts from the point [1,0,1]^*T*^ with a period of 2*π* s.

Figure 10F shows the states for all robots in the step response test. Green dashed curves show desired states from the planner, and black dashed and solid curves show the response of the SoAG robot with the arm lifted. Blue dashed and solid curves denote the response of the robot when the arm is down. Note that the time is synchronized solely in the visualization for better comparison. The results show that Quad has a faster rising time compared to the other four cases in both $x_{W}$ and $y_{W}$ directions due to reduced weight. However, the response along $z_{W}$ axis is similar for all robots since it aligns with the thrust direction. On the other hand, the SoAG robot has a very close step response in different arm and gripper states, which demonstrates that the rigid arm and soft gripper designs do not compromise the flyability of the aerial robot. A similar conclusion can be made in the planar circle test as shown in Figure 10G,H, which visualizes positions and velocities on $x_{W}$ and $y_{W}$ axis. The green dashed curve shows the desired states for the circle trajectory, with a jump on the velocity along $y_{W}$ in the beginning. Due to the discontinuity, the SoAG robot has a slower converging rate compared to Quad in the first 2 s. All robots have good position and velocity tracking on $y_{W}$ axis afterwards. The desired trajectory is smooth in the $x_{W}$ direction, thus, all robots have good tracking performance throughout the test.

## 4 Discussion

In this work, we focus on addressing the challenge of catching a aerial micro-robot in mid-air using another MAV equipped with a soft gripper. Specifically, we introduce a gripper design based on soft actuators that keep a horizontally straight shape with a single fixture and maintain sufficiently compliance when bending. To enable horizontal grasping, we further develop an open-source MAV equipped with the end-effector and onboard pneumatic regulation named Soft Aerial Gripper (SoAG). The hardware design is introduced, as well as the dynamic modeling and control. We present a planning method based on piecewise polynomial optimization to catch the flying micro-robots without generating aerodynamic disturbances detrimental to the target’s stability.

Experimental results show the low-cost soft gripper, powered by light weight air pumps that are onboard the robot, has fast opening and closing responses as compared to commercially available end-effectors. Static grasping tests study the soft gripper’s robustness in capturing aerial micro-robots under the influence of aerodynamic disturbances. We experimentally demonstrate the feasibility of using the SoAG robot to catch a hovering micro-robot and return with the target. The free flight performance of the SoAG robot is studied against a conventional quadrotor and in different gripper and payload status to validate the design. To the authors’ knowledge, the SoAG robot is the first MAV to demonstrate the feasibility of catching a flying micro-robot with a soft gripper. The robot can be used in search and rescue of aerial robots or seize unidentified flying targets without damage. In the meantime, the robot can move fragile objects as a conventional aerial gripper, with potential applications in aerial transportation and construction.

With the introduction and validation of the SoAG robot in this paper, several directions for future work can be enabled. For instance, small aerial robots have limited flight time so one way to improve their energetics besides other means currently at the forefront of research Karydis and Kumar (2017) would be to be caught and released in mid-air safely *via* larger aerial robots. Another direction of interest includes safe multi-robot co-manipulation for transportation and assembly. The compliance afforded by the soft gripper can help account for positioning errors of the robots and thus help relax some of the optimization constraints in aerial co-manipulation. Furthermore, at the current stage of development, the flying target’s weight in this work is very small. To enable grasping and transportation of heavier objects, we plan to upgrade the MAV hardware and incorporate the target’s mass onto the system’s modeling as in Mellinger et al. (2011), as well as robust or adaptive controlling methods to address changes in mass and inertia. While the aerial vehicle has a maximum payload of 1.2 kg, the proposed soft gripper can only grasp objects with masses up to 280 g. To scale up the solution, we plan to strengthen the grasping capacity by using stronger materials and pneumatic actuation. Third, the project uses the basic on-off control for the gripper with constant inflating rates. It is of interest to incorporate feedback control of the soft end-effector as in Mucchiani et al. (2022). Fourth, we aim to deploy the robot in outdoor or confined environments without a motion capture system, thus enabling vision guidance as in Kim et al. (2016) and impact resilience as in Liu and Karydis (2021b). Lastly, it is of interest to discover the possibility of combining aerial-ground robots by including pneumatic legged mobility as in Liu et al. (2020); Liu and Karydis (2021a).

## Data Availability

The files for developing the robot can be found in the repository https://github.com/UCR-Robotics/SoAG. The raw data supporting the conclusion of this article will be made available by the authors. Inquiries can be directed to the corresponding author.
